# Chemical composition, immunostimulatory, cytotoxic and antiparasitic activities of the essential oil from Brazilian red propolis

**DOI:** 10.1371/journal.pone.0191797

**Published:** 2018-02-01

**Authors:** Ângela Sena-Lopes, Francisco Silvestre Brilhante Bezerra, Raquel Nascimento das Neves, Rodrigo Barros de Pinho, Mara Thais de Oliveira Silva, Lucielli Savegnago, Tiago Collares, Fabiana Seixas, Karine Begnini, João Antonio Pêgas Henriques, Mariana Roesch Ely, Luciane C. Rufatto, Sidnei Moura, Thiago Barcellos, Francine Padilha, Odir Dellagostin, Sibele Borsuk

**Affiliations:** 1 Centro de Desenvolvimento Tecnológico (CDTEc), Universidade Federal de Pelotas (UFPel), Campus Capão do Leão, Capão do Leão, Rio Grande do Sul, Brazil; 2 Departamento de Tecnologia, Instituto de Biotecnologia, Universidade de Caxias do Sul, Caxias do Sul, Rio Grande do Sul, Brazil; 3 Instituto de Tecnologia e Pesquisa (ITP), Universidade de Tiradente, Aracaju, Sergipe, Brazil; National Taiwan University, TAIWAN

## Abstract

Most studies of Brazilian red propolis have explored the composition and biological properties of its ethanolic extracts. In this work, we chemically extracted and characterized the essential oil of Brazilian red propolis (EOP) and assessed its adjuvant, antiparasitic and cytotoxic activities. The chemical composition of EOP was analyzed using gas chromatography with mass spectrometry (GC-MS). EOP was tested for in vitro activity against *Trichomonas vaginalis* (ATCC 30236 isolate); trophozoites were treated with different concentrations of EOP (ranging from 25 to 500 μg/mL) in order to establish the MIC and IC_50_ values. A cytotoxicity assay was performed in CHO-K1 cells submitted to different EOP concentrations. BALB/c mice were used to test the adjuvant effect of EOP. The animals were divided in 3 groups and inoculated as follows: 0.4 ng/kg BW EOP (G1); 50 μg of rCP40 protein (G2); or a combination of 0.4 ng/kg BW EOP and 50 μg of rCP40 (G3). Total IgG, IgG1 and IgG2a levels were assessed by ELISA. The major constituent compounds of EOP were methyl eugenol (13.1%), (E)-β-farnesene (2.50%), and δ-amorphene (2.3%). Exposure to EOP inhibited the growth of *T*. *vaginalis*, with an IC_50_ value of 100 μg/mL of EOP. An EOP concentration of 500 μg/mL was able to kill 100% of the *T*. *vaginalis* trophozoites. The EOP kinetic growth curve showed a 36% decrease in trophozoite growth after a 12 h exposure to 500 μg/mL of EOP, while complete parasite death was induced at 24 h. With regard to CHO-K1 cells, the CC_50_ was 266 μg/mL, and 92% cytotoxicity was observed after exposure to 500 μg/mL of EOP. Otherwise, a concentration of 200 μg/mL of EOP was able to reduce parasite proliferation by 70% and was not cytotoxic to CHO-K1 cells. As an adjuvant, a synergistic effect was observed when EOP was combined with the rCP40 protein (G3) in comparison to the administration of each component alone (G1 and G2), resulting in higher concentrations of IgG, IgG1 and IgG2a. EOP is constituted by biologically active components with promising antiparasitic and immunostimulatory activities and can be investigated for the formulation of new vaccines or trichomonacidal drugs.

## Introduction

Currently, the Brazilian propolis is classified in 13 different types, according to its physical-chemical properties, botanical origin and geographic area where propolis can be collected. Initially, Park et al. [1] classified propolis samples from different Brazilian regions in 12 groups. Afterwards, a 13^th^ type of propolis with peculiar chemical composition was found in hives located alongside the coast and mangroves in the Brazilian northeast and was called Brazilian red propolis (BRP) [2]. In the last decade, the chemical composition of BRP has been revealed and related to its bioactivities. The main chemical components found in BRP are classified as isoflavones, flavones, flavonols, aurones, chalcones, pterocarpans and xanthones groups [3–6]. The botanical origin of BRP was identified as resinous exudates of *Dalbergia ecastophyllum* [7], however, likely a second plant species participates as one of the main sources of resins for BRP [5].

Due to the variety of chemical compounds of BRP incorporated within the resin mixture, especially phenolic compounds, many biological activities have been reported like antioxidant [2,8], antibacterial [9,10], fungicide [11], antiparasitic [10,12], antineoplasic [13,14], anti-caries [15], cytotoxic [2,10], anti-inflamatory [16,17], antinociceptive [18] and was also beneficial to the wound healing process [19].

Several experiments and clinical investigations have shown the antiparasitic and adjuvant properties of the hydroalcoholic extract of propolis (HEP). HEP of different types of propolis showed antiparasitary efficacy against *Giardia lamblia* [20,21], *Toxoplasma gondii* and *Trichomonas vaginalis* [22]. It is important to highlight that *T*. *vaginalis* is the etiological agent of trichomoniasis, the most common non-viral, sexually transmitted disease (STD) in the world [23]. In addition, metronidazole-resistant T. vaginalis has been implicated in an increasing number of refractory cases, which are usually treated with increased doses of the drug, leading to an increase in the occurrence of side effects [24]. Clearly, alternative curative therapies are needed, and propolis emerges as a natural product for alternative drugs.

Moreover, HEP has been used to formulate veterinary vaccines against bacterial or viral diseases, once some phenolic compounds has presented immunomodulatory activity [25–27]. As adjuvant, propolis confers to the vaccinal formulation desirable properties, such as reduced viscosity, good stability, low reactogenicity, low toxicity, and is able to induce a robust humoral and cellular response [26].

As aforementioned, most of the previous studies describing the biological activities of BRP uses ethanol as the extraction solvent, and the extraction method influences directly the yield and selectivity for some compounds [10,28]. Volatile compounds like those found in essential oils are in low concentrations in propolis, but play an important role by contributing to propolis pleasant aroma and its biological activities. Researchers have applied various methods to obtain propolis volatiles such as distilllation-extraction or hydrodistillation [29].

On the other hand, studies focusing on essential oils of propolis volatiles are relatively scarce, most of them dealing with antimicrobial properties against fungus, Gram-positive and Gram-negative bacteria [29]. Volatiles of propolis from stingless bees were able to stimulate the immune system of elderly patients by increasing their natural killer cell activity [30]. Essential oils of Chinese propolis inhibited the proliferation of human colorectal cancer cells by inducing cell cycle arrest and apoptosis [31]. However, to the best of our knowledge, there are no studies describing the chemical composition or the biological activities of the essential oil derived from the Brazilian red propolis (EOP). Herein, the chemical composition of the EOP is showed. Still, in vitro trichomonacidal and cytotoxic activities, and the adjuvant action of EOP in a recombinant vaccine are assessed.

## Materials and methods

### Red propolis sample collection and essential oil preparation

Red propolis was collected from the municipality of Brejo Grande, located in the state of Sergipe, in northeastern Brazil (S 10°28'25” and W 36°26'12”). Samples were collected in September 2011 and were maintained frozen at −20 °C until use. For the preparation of the essential oil, conventional hydrodistillation was carried out using a Clevenger-type apparatus. Briefly, 380 g of BRP was transferred to a volumetric flask, and 2 L of distilled water was added. A heating mantle was placed onto the Clevenger apparatus. The essential oil was extracted after boiling the mixture for a time period of one hour. The essential oil obtained was collected in amber vials and stored at 4 °C until use. The total yield obtained was approximately 0.25%.

#### Gas chromatography–mass spectrometry (GC-MS)

Chemical composition analysis was performed using a gas chromatograph coupled with a mass-selective detector (Hewlett Packard 6890/MSD5973, Palo Alto, USA) equipped with the HP ChemStation software and the Wiley 275 library. A capillary column composed of HP-INNOWax fused silica (30 m × 250 μm) of 0.50 μm film thickness (Hewlett-Packard, Palo Alto, USA) was used for the study. The temperature program used was as follows: the column temperature started at 40 °C (held for 8 min) and was increased to 180 °C at 3 °C/min and then to 230 °C (held for 20 min) at 20 °C/min; the injector was at a temperature of 250 °C and the interface at 280 °C. The split ratio was 1:100, helium was the carrier gas (56 kPa), the flow rate was 1 mL/min, the ionization energy was 70 eV, and the injected volume was 0.4 mL.

### Anti-*Trichomonas vaginalis* activity assay

The *T*. *vaginalis* 30236 isolate used for evaluating antiparasitic activity in this study was obtained from the American Type Culture Collection (ATCC). Trophozoites were axenically cultured in trypticase-yeast extract-maltose (TYM) medium (without agar; pH = 6.0) supplemented with 10% sterile bovine serum (inactivated at 56 °C) and incubated at 37 °C [32]. Cultures with 95% viability, as confirmed through observation of motility, morphology, and trypan-blue exclusion (0.4%) assay under the light microscope at 400X magnification, were considered as alive and were used for evaluating the activity of EOP.

All assays were performed in 96-well microtiter plates (Cral^®^). Parasites were seeded at an initial density of 2.6 × 10^5^ trophozoites/mL of TYM and incubated in the presence of EOP. Three experimental controls were also performed: parasites only, the vehicle used for solubilization of EOP (0.6% DMSO) and 100 μM MTZ (metronidazole; Sigma-Aldrich) (as a positive control). The microculture plates were incubated at 37 °C with 5% CO_2_ for 24 h. Subsequent to the incubation, a preparation containing trophozoites and trypan blue (0.4%) in a 1:1 ratio was prepared and counted in a Neubauer chamber. Cultures with 95% viability were utilized for the assay.

To determine the minimum inhibitory concentration (MIC), different concentrations of EOP (25, 50, 100, 200, 300, 400, and 500 μg/mL) were tested as described by Hübner et al. [33], with some modification. Parasite pellets were used to establish the MIC as well as concentrations below and above the MIC value. Samples used as controls were inoculated in fresh TYM medium at 37 °C. Parasites were counted in a Neubauer chamber with trypan blue every 24 h for 96 h to confirm MIC. The viability of trophozoites was assessed by the exclusion of the trypan blue dye as well as by motility and morphology. The IC_50_ (half the maximum inhibitory concentration) values were determined at the same concentrations used for the MIC test.

A kinetic growth curve was constructed in order to obtain a more comparable activity profile of EOP activity against *T*. *vaginalis*. The viability of trophozoites was observed under a light microscope 96 h after incubation with EOP at the required MIC. Growth analysis was performed at 1, 6, 12, 24, 48, 72, and 96 h by the trypan blue (0.4%) exclusion method and by the evaluation of motility and morphology. All assays were performed independently at least three times in triplicate, and the results were expressed as the percentage of viable trophozoites compared to untreated parasites.

For the Anti-*Trichomonas vaginalis* activity assay, statistical analysis was conducted using one-way analysis of variance (ANOVA), and a probability value of *p* < 0.05 was considered significant. Tukey’s test was utilized to identify significant differences between the means of different treatments. GraphPad Prism version 5.0 for Windows (GraphPad Software, USA) was used for statistical analysis.

#### Cytotoxicity assay

Chinese hamster ovary (CHO-K1) cells were obtained from the Rio de Janeiro Cell Bank (PABCAM, Federal University of Rio de Janeiro, RJ, Brazil) and cultured as a monolayer in Dulbecco’s Modified Eagle’s Medium (DMEM) (Vitrocell Embriolife) supplemented with 10% fetal bovine serum (FBS) (Vitrocell Embriolife), 1% L-glutamine, and 1% penicillin/streptomycin. The cells were grown at 37 °C in an atmosphere of 95% humidified air and 5% CO_2_.

The post-treatment proliferation of the CHO-K1 cell line was determined by measuring the reduction of soluble 3-(4,5-dimethylthiazol-2-yl)-2,5-diphenyl tetrazolium bromide (MTT, Sigma-Aldrich) to water-insoluble formazan. Cells were seeded in 96-well plates at a density of 2 × 10^4^ cells per well in 100 μL volumes and grown at 37 °C in a 5% CO_2_ atmosphere for 24 h before they were used in the cell viability assay. The cells were then treated with 31.25, 62.5, 125, 250 and 500 μg of EOP for 24 h. DMSO was used as vehicle control (VC), untreated cells were used as a negative control and 100 μM of metronidazole (MTZ) was used as a positive control. Following the incubation, 20 μL of MTT (Sigma-Aldrich) was added to each well and the cells were incubated for an additional period of 3 h at 37 °C. Differences in the total cellular metabolism were detected at a wavelength of 492 nm by using a microplate reader. The inhibition (%) of cell proliferation was determined as follows: inhibitory growth = (1 − Abs_492_ treated cells / Abs_492_ control cells) × 100%. The IC_50_ was calculated using GraphPad Prism version 5.0 for Windows (GraphPad Software, USA). All observations were validated by at least three independent experiments in triplicate.

### Adjuvant assay

#### Experimental animals

Six- to eight-week-old female BALB/c mice were obtained from the Central Animal Facility of the Federal University of Pelotas, where the immunization assays were also conducted. All mice were kept in cages containing wood shavings and bedding and had free access to water and a maintenance diet *ad libitum*. A 12-h light/dark cycle at room temperature (21 ± 2 °C) was also maintained. All experiments were performed in compliance with the procedures of the Brazilian College of Animal Experimentation (COBEA). The Ethics Commission on Animal Experimentation (CEEA) of Pelotas Federal University (UFPel) approved the project (number 2422). All efforts were made to minimize animal suffering.

#### Antigen expression and purification

The recombinant endoglycosidase CP40 (rCP40), derived from *C*. *pseudotuberculosis*, was used as an antigen for the adjuvant assay. The pAE/*cp40* recombinant plasmid previously constructed by our group [34] was transformed into the *Escherichia coli* BL21 (DE3) Star strain by the heat shock method. The induction of expression was performed by adding 1 mM of IPTG followed by incubation at 37 °C for 3 h with constant agitation. Western blotting using a monoclonal anti-6x-His-tag antibody conjugated to peroxidase (Sigma-Aldrich) was used to confirm rCP40 expression. A sepharose-nickel column, HisTrapTM (GE Healthcare), was used for purification of the recombinant protein. Purity was determined by 12% SDS-PAGE, and the concentration was measured using a BCA kit (PIERCE).

#### Immunization assay and blood sample collection

For the immunization assay, the mice were divided into the following three groups of six animals each: (a) G1: a negative control group immunized with 0,4 ng/kg BW EOP; (b) G2: inoculated with only 50 μg of rCP40 protein; and (c) G3: the test group inoculated with 0,4 ng/kg BW EOP plus 50 μg of rCP40. All doses were prepared in a final volume of 200 μL using sterile saline solution (0.9% NaCl) as a vehicle and administered via the intramuscular route. The animals were immunized with two doses administered 21 days apart. Animals were bled at days 0, 21, and 42 post-immunization to determine the antibody levels. Blood was collected from the retro-orbital sinus with Pasteur pipettes. All blood collections were performed under anesthesia with 10% ketamine (1 mL/kg) and 2% xylazine (0.1 mg/kg) (Agener União Saúde Animal, Brazil). After coagulation, the blood was centrifuged at 1,500 g for 15 min. The serum was removed and stored at −20 °C.

#### Assessment of antibody production

Serum samples from the immunized mice were used in immunoassays (indirect ELISA) for the quantification of total IgG, IgG1 and IgG2a anti-rCP40 antibodies. Polystyrene 96-well plates (Maxisorp-Nunc) were coated with 100 μL of a bicarbonate-carbonate buffer (pH 9.8) containing 0.1 μg/well of rCP40 protein. Plates were incubated for 18 h at 4 °C and washed 3 times with PBS-T (PBS 1X, pH 7.4, 0.1% Tween 20). The plates were then incubated with 200 μL/well of 5% skim milk in PBS for 2 h at 37 °C, for blocking purposes. A three-step wash was then performed. All serum samples obtained from mice were diluted in PBS (v/v 1:50), and 100 μL of each diluted sample was added to the plates in duplicate. Plates were incubated for 1 h at 37 °C. After washing three times with PBS-T, 100 μL/well of anti-mouse IgG conjugated to horseradish peroxidase (Sigma-Aldrich) was added (1: 5,000 in PBS-T) to the plates in order to detect total IgG prior to the revelation step. In plates designated for the detection of IgG1 and IgG2a, 100 μL/well of either goat anti-mouse IgG1 (1: 5,000 in PBS-T) or goat anti-mouse IgG2a (1: 2,000 in PBS-T) was added. The plates were incubated at 37 °C for 1 h and then washed three times with PBS-T, subsequent to which 100 μL/well of anti-goat IgG conjugated to horseradish peroxidase (Sigma-Aldrich) was added (1: 5,000 in PBS-T). The plates were re-incubated for 1 h at 37 °C and then washed five times with PBS-T. The reaction was developed by the addition of 100 μL/well of the substrate-chromogen solution [*o*-phenylenediamine dihydrochloride; OPD tablets (Sigma-Aldrich) in 0.4 mg/mL phosphate-citrate buffer containing 0.04% of 30% hydrogen peroxide, pH = 5.0] and incubation at room temperature in the dark for 15 min, and it was stopped by the addition of 50 μL/well of H_2_SO_4_ solution (4N). Optical density (OD) was determined using a microtiter-plate reader (Microplate Reader Mindray MR–96A) set at 492 nm.

Data were analyzed using the software GraphPad Prism version 5.0 for Windows (GraphPad Software, USA). Differences in IgG levels between the groups were analyzed using one-way ANOVA followed by Tukey's test for multiple comparisons. The results were considered statistically significant if p-value <0.05.

## Results

### EOP chemical components

GC-MS analysis of the red propolis extract enabled the identification of certain compounds present in the essential oil. A list of these constituents is presented in Table 1. GC-MS is a reliable method, indispensable in several areas where the analysis of complex mixtures and unambiguous identification is required, and since volatile compounds are well identified with GC-MS, this method is suitable for analysis of essential oils [35,36]. Using GC-MS, our study was able to highlight methyl eugenol (13.1%), (E)-β-farnesene (2.5%), δ-amorphene (2.3%), α-cubebene (1.9%), and β-caryophyllene (1.5%) as the major components of the extract.

**Table 1:** Volatile compounds identified by gas chromatography followed by mass spectrometry (GC-MS) of the Brazilian red propolis essential oil.

Compound	Area (%)	LRI obtained	LRI reference
α-Pinene	0.2	908	907 [37]
α-Cubebene	1.9	1351	1351[37,38]
(-)-Cyperene	0.2	1401	1400 [39]
Methyl eugenol	13.1	1407	1408 [40]
α-Gurjunene	0.5	1412	1412 [41]
β-Caryophyllene	1.5	1422	1422 [42]
trans-α-Bergamotene	0.6	1438	1438 [43]
(E)-β-Farnesene	2.5	1459	1459 [37]
γ-Muurolene	0.2	1479	1479 [44]
α-Curcumene	0.1	1485	1484 [45]
Selinene	0.7	1490	1490 [46]
β-Bisabolene	0.9	1506	1506 [47]
δ-Amorphene	2.3	1514	1514 [48]

### Anti-*Trichomonas vaginalis* activity assay

**Fig 1:**
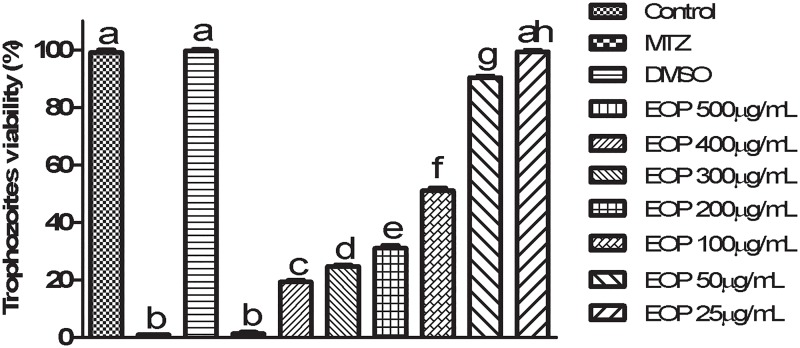
*Trichomonas vaginalis* 30236 isolate; MIC and IC_50_ after 24-hour-treatments with EOP at 25, 50, 100, 200, 300, 400, 500 μg/mL. Data represent mean ± standard deviation of at least three experiments, all in triplicate. Different letters indicate a significant difference (p < 0.05).

**Fig 2:**
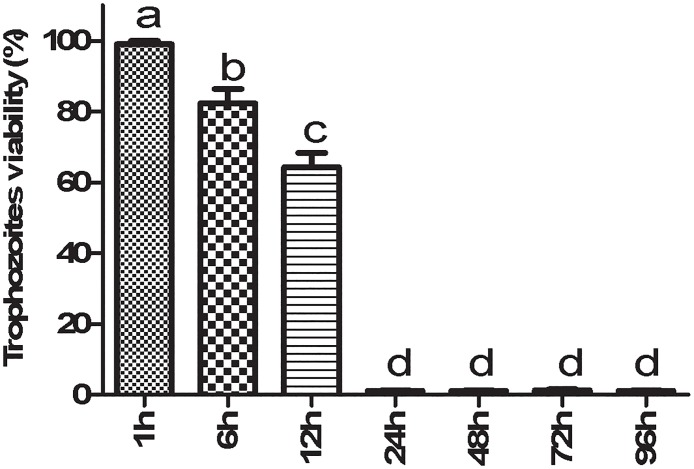
Kinetic growth curve of the *Trichomonas vaginalis* 30236 isolate after exposure to EOP at 500 μg/mL for 1 h, 6 h, 12 h, 24 h, 48 h, 72 h and 96 h. Data represent mean ± standard deviation of at least three experiments, all in triplicate. Different letters indicate a significant difference (p < 0.05).

Analysis of the EOP MIC data revealed that at a 500 μg/mL concentration, 100% death of trophozoites was observed. After 24 h of exposure, EOP demonstrated optimal anti-*T*. *vaginalis* activity at a concentration of 500 μg/mL with an IC_50_ value of 100 μg/mL (Fig 1). The negative control and DMSO control showed positive motility and did not stain with Trypan blue (0.4%), whereas the positive control stained blue and exhibited negative motility at the 24 h time point in all assays performed. When the kinetic growth curve was analyzed, it was observed that the exposure to 500 μg/mL of EOP reduced the growth of trophozoites by 36% at 12 h and induced complete parasite death at 24 h (Fig 2).

### Cytotoxicity assay

**Fig 3:**
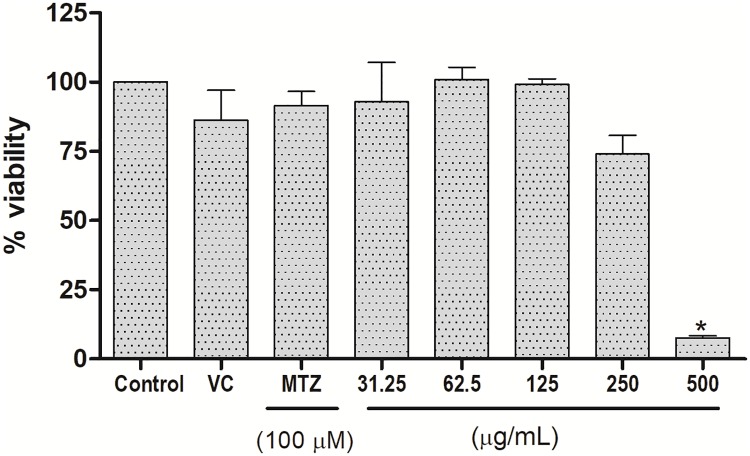
CHO-K1 cell viability after treatment with different concentrations of EOP. Cell proliferation in CHO-K1 was investigated by the MTT assay, and metronidazole was used as a positive control. Data are expressed as the mean ± SEM of the viabilities of CHO-K1 cells in three independent experiments. (*) indicates a difference between the treatments in CHO-K1 cells. The differences were considered significant at p < 0.05. VC = vehicle control; MTZ = metronidazole.

EOP treatment also inhibited proliferation of the CHO-K1 cell line; however, this cytotoxicity was only significant at 500 μg/mL. No significant cytotoxicity was observed up to 250 μg/mL in growth inhibition of CHO-K1 cells after 24 h of treatment (Fig 3). The in vitro cytotoxicity activity of EOP showed IC_50_ values of 266 and 100 μg/mL in CHO-K1 cells and trophozoites, respectively, which may indicate selectivity of the EOP treatment at concentrations up to 250 μg/mL. The results enumerated above demonstrate that EOP is an important resource for future drug development, and its therapeutic potential against various infectious entities merits further study and analysis.

### Adjuvant assay

**Fig 4:**
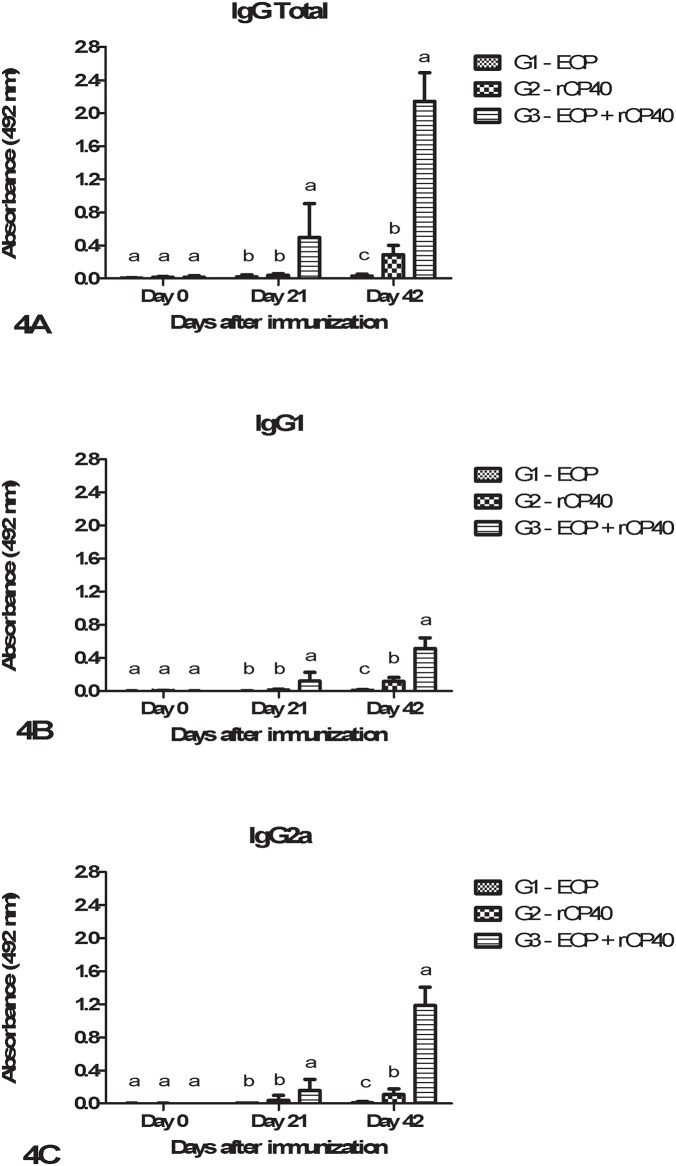
Anti-rCP40 total IgG (4A), IgG1 (4B) and IgG2a (4C) levels in immunized mice treated with EOP and recombinant endoglycosidase CP40 from *C*. *pseudotuberculosis* (rCP40), either separately or together. Measurements were taken on day 0 and then 21 and 42 days post-immunization. Data represent means ± standard deviations of IgG levels in the sera collected from 6 animals/group. Different letters in the same experimental day indicate a significant difference (p < 0.05).

Fig 4 illustrates the results obtained from the ELISA assay designed for analyzing the production of anti-rCP40 total IgG, IgG1, and IgG2a antibodies. The results demonstrate that animals from group G1 were unable to induce production of IgG and its isotypes. In group G2, increased total IgG, IgG1, and IgG2a levels were detected on day 42, subsequent to the administration of two doses of rCP40. Additionally, it was observed that the association of EOP with rCP40 (G3) leads to the induction of higher levels of IgG and its isotypes. When compared with groups G1 and G2, group G3 was observed to present a significantly higher increase (p < 0.05) in total IgG total as well as isotypes IgG1 and IgG2a at days 21 and 42 after the first immunization (Fig 4A, 4B and 4C); this is demonstrative of the adjuvant effect of EOP. The day-42 values clearly demonstrate that the association of EOP with rCP40 was able to increase the levels of total IgG, IgG1 and IgG2a by approximately 7.4, 4.4 and 10.8 fold, respectively, in comparison to the administration of rCP40 alone. Moreover, group G3 shows more pronounced IgG2a production compared to the production of IgG1 at day 42.

## Discussion

Although some Brazilian propolis essential oils, mainly those derived from green propolis, have been characterized previously [36,49,50], this is the first study to undertake the characterization of BRP essential oil. Only the ethanolic and methanolic extracts of BRP have been tested being isoflavones the main isolated compounds [18,51,52]. Thus, we hypothesized that the essential oil (EO) of BRP could provide some volatile compounds with important biological properties, which are probably lost or eliminated in the other extraction methods. In fact, methyl eugenol, a phenylpropene, was the major volatile component isolated from EOP and most of the other isolates were classified as sesquiterpenes. Along similar lines, it is known that the Brazilian green propolis also has a predominance of sesquiterpenes. Nerolidol, β-caryophyllene, spathulenol, and δ-cadinene have been identified as other major volatile components of green propolis [29]. Studies have revealed that EO constituents make up a diverse family of low-molecular-weight organic compounds with extreme variations in biological activity. Based on their chemical structure, the active compounds of EOs can be divided into four major groups: terpenes, terpenoids, phenylpropenes, and “others” [53].

Some of the volatile compounds present in EOP have already been credited with having antiparasitic activity. These chemical entities include β-caryophyllene, α-pinene, α-bergamotene, and (E)-β-farnesene [54,55]. Additionally, methyl eugenol isolated from the essential oil extracted from the plant *Agastache rugosa* showed significant nematicidal activity against *Meloidogyne incognita* [56]. Methyl eugenol is a specific constituent of Brazilian red propolis, among another types of propolis, which was revealed for the first time in 2008 [57]. Therefore, in this work, we hypothesized that methyl eugenol, a phenylpropene with the major concentration in EOP and the sesquiterpenes may be responsible for the antiparasitic activity against *T*. *vaginalis* exhibited here. However, further studies should be performed with the isolated constituents of EOP to identify the specific antiparasitic agents.

In spite of the anti-*T*. *vaginalis* activity, a higher cytotoxicity level was observed at the EOP concentration that presented 0% viability of trophozoites after 24 h of exposure (500 μg/mL) compared to a lower concentration (200 μg/mL), which was not toxic for CHO-K1 cells but reduced parasite proliferation by 70%. Therefore, it is hypothesized that cytotoxicity could be a limiting factor for the parenteral use of EOP, but its use in topical forms for the treatment of trichomoniasis, through topical vaginal application, appears to be an interesting alternative. It is well known that topical application allows for the delivery of higher drug concentrations to the vaginal epithelium at lower treatment doses [58].

A previous study has evaluated the effects of compounds with guaiacyl and syringyl structures (as degradation products of lignin) and natural aromatic acids and their aldehydes on *T*. *vaginalis* and found that the tested compounds exhibited antiparasitic activity with MIC values ranging from 10 to 200 μg/mL. Isoeugenol, a phenylpropene generated by a propenyl-substituted guaiacol, was the most potent effector against *T*. *vaginalis* with a MIC value of 10 μg/mL [59], which is much lower than MIC found here for EOP. Since EOP is a complex mixture containing mainly methyl eugenol, a phenylpropene with similarities with isoeugenol in chemical structure, and sesquiterpenes in low concentrations, it is expected a different biological result in comparison to the use of each isolated compound.

In addition, some studies have revealed that red propolis extracts from different geographical regions have significant potential as antiparasitic agents. The ethanolic extract of red propolis obtained from Nigeria, when tested against *Trypanosoma brucei*, demonstrated potent antiparasitic activity in both sensitive and resistant strains [60]. In another study, four samples of ethanolic extracts of Brazilian propolis were tested against *Leishmania amazonensis*. Three extracts of green propolis and one of red propolis were tested, and all of them were capable of reducing parasite loads in infected macrophages. Nevertheless, the extract obtained from BRP showed maximal activity against *L*. *amazonensis* while being non-toxic to the macrophage cultures [61].

Recently, the antiparasitic activity of BRP has attracted the attention of researchers. Polymeric nanoparticles of the BRP extract and the ethanolic extract of BRP have demonstrated anti-leishmanial activity against *L*. *(V*.*) braziliensis*, with IC_50_ values of 31.3 μg/mL and 38.0 μg/mL, respectively, demonstrating the potential of BRP in the composition of pharmaceuticals for leishmaniasis therapy [12]. Additionally, when ethanolic extracts from various types of green, brown and red propolis from different regions of Brazil were tested against *Trypanosoma cruzi* Y-strain epimastigotes, an inhibitory effect on parasite growth was observed in the first 24 h for all the types of propolis. However, only the ethanolic extracts of BRP presented a persistent *T*. *cruzi*-inhibitory effect after 96 h, suggesting red propolis as a potential alternative for the therapeutic treatment of Chagas disease [10].

In this study, we also confirmed that EOP has the potential to be used as an adjuvant in a veterinary vaccine formulation. In our study, a recombinant endoglycosidase CP40 (rCP40), encoded by the *cp40* gene of *C*. *pseudotuberculosis*, was used as the antigen as its antigenic properties have been previously confirmed [34,62]. Our results confirmed that association of the rCP40 protein with EOP improves the immune response to this target compared to the administration of either of these components alone. We observed that the combination of the rCP40 with EOP led to IgG absorbances higher than those found by other researchers using traditional adjuvants such as saponin or Freund’s complete adjuvant [34]. Herein, we used for the first time EOP as an adjuvant. In fact, this is the first study to evaluate the effect of BRP as a vaccinal adjuvant.

Compared to the administration of rCP40 alone, a combination of the recombinant protein with EOP was able to induce increases of 7.4-, 4.4- and 10.8-fold higher levels of total IgG, IgG1, and IgG2a, respectively. One of the most important challenges currently facing adjuvant research is the search for the ‘perfect mix’: an optimal, safe formulation whose different components are not only additive but also synergistic in nature, and whose administration gives rise to the desired robust immune response [63]. In this study, we observed a synergistic effect when EOP was associated with rCP40. Along similar lines, another recent study has reported that the use of 3% palm oil with a bacterin of recombinant *E*. *coli* carrying the Omp40 protein resulted in an enhancement of IgG levels and of CD4^+^ and CD8^+^ T-cell responses, while administration of the bacterin without adjuvant failed to stimulate the humoral and cell-mediated immunities [64].

Until 42 days after immunization, the IgG2a production levels were higher than those observed for IgG1, which is indicative of a T-helper 1 cell (Th1) response. In mice, Th1 cells aid in the activation of macrophages and cytotoxic T-cells and in the production of opsonizing and complement-activator immunoglobulin isotypes, such as IgG2a. Hence, it is widely believed that the primary function of Th1 cells is the generation of immunity against intracellular pathogens [34,65]. Vaccine formulations that enhance Th1 levels and CD8^+^ T-cell responses are highly sought after as they are effective for immunization against pathogens for which there are no currently licensed vaccines (examples: HIV-AIDS, malaria, and tuberculosis) [66]. EOP has the potential to serve as an excellent adjuvant for immunization purposes. Once propolis is considered both safe and non-toxic when administered to animals or humans [26], EOP could be a good adjuvant to be used in important vaccines, and further investigations using this substance should be conducted.

The immunostimulatory effect of EOP may be attributed to the high levels of methyl eugenol (13.1%). Eugenol is a phenylpropene that is commonly found in essential oils; previous studies have established that eugenol stimulates the immune response in mice by promoting the T-cell response and natural killer activity [67]. It is also possible that sesquiterpenes could be involved in the adjuvant effect of BRP essential oil as it is known that they also activate the immune system by influencing local inflammation and promoting phagocytosis [68].

Most of the studies involving the effect of BRP on the immune system are recent and have extensively investigated the in vivo and in vitro effects of hydroalcoholic extracts of BRP on the inflammation process [16–19,69,70]. BRP reduces nitric oxide levels and diminishes the levels of some pro-inflammatory cytokines, chemokines and genes associated with inflammatory signaling in macrophages [16,17]. Additionally, BRP modulates neutrophil migration by interfering in rolling and adhesion processes through reduction of the levels of TNF-α, IL-1β, CXCL1/KC and CXCL-2/MIP-2 and reduction of calcium influx [70]. In vivo, BRP has been seen to promote protective effects against ulcerative colitis in a rat model, reducing gross and histological inflammatory lesions and decreasing the levels of myeloperoxidase and iNOS in colon tissue [69]. Moreover, mice treated orally with BRP showed improved cutaneous wound healing through faster wound closure, reduction of inflammatory infiltrate, and downregulation of the transcription factor pNF-κB and the inflammatory cytokines TGF-β, TNF-α and IL-6 [19]. Antinociceptive activity of BRP towards neurogenic and inflammatory pain in mice was also observed without emotional and motor side effects [18].

The immunomodulatory properties of propolis seem to be contradictory since propolis sometimes acts in the inhibition, as in the anti-inflammatory properties, and other times in the stimulation of the immune system, as in the adjuvant properties. This phenomenon is probably caused by the chemical complexity of propolis as a result of the synergism among the components, which can be varied according to the methods of extraction of the bioactive compounds [71].

## Conclusion

The EOP was observed to exhibit a promisingly high level of antiparasitic activity, with cytotoxicity seen at higher concentrations of EOP. However, further studies using lower concentrations of EOP for topical application should be performed with the aim of reducing cytotoxicity levels and achieving potent localized action in the treatment of trichomoniasis. Our study also demonstrated the potential use of EOP as a vaccine adjuvant since the administration of EOP along with rCP40 was seen to increase IgG production. The results of this study shed light on several new properties of EOP and underline the importance of exploring its potential as an additional option for the treatment of trichomoniasis and as a vaccine adjuvant. Furthermore, studies dedicated to revealing the potential and the importance of EOP in other biological activities are encouraged, due to EOP chemical complexity.

## References

[1] Park YK, Alencar SM, Aguiar CL. Botanical Origin and Chemical Composition of Brazilian Propolis. J Agric Food Chem. 2002;50: 2502–2506. doi: 10.1021/jf011432b 11958612 10.1021/jf011432b

[2] Alencar SM, Oldoni TLC, Castro ML, Cabral ISR, Costa-Neto CM, Cury JA, et al Chemical composition and biological activity of a new type of Brazilian propolis: Red propolis. J Ethnopharmacol. 2007;113: 278–283. doi: 10.1016/j.jep.2007.06.005 17656055 10.1016/j.jep.2007.06.005

[3] Awale S, Li F, Onozuka H, Esumi H, Tezuka Y, Kadota S. Constituents of Brazilian red propolis and their preferential cytotoxic activity against human pancreatic PANC-1 cancer cell line in nutrient-deprived condition. Bioorganic Med Chem. 2008;16: 181–189. doi: 10.1016/j.bmc.2007.10.004 17950610 10.1016/j.bmc.2007.10.004

[4] Li F, Awale S, Tezuka Y, Kadota S. Cytotoxic constituents from Brazilian red propolis and their structure-activity relationship. Bioorganic Med Chem. 2008;16: 5434–5440. doi: 10.1016/j.bmc.2008.04.016 18440233 10.1016/j.bmc.2008.04.016

[5] López BG-C, Schmidt EM, Eberlin MN, Sawaya ACHF. Phytochemical markers of different types of red propolis. Food Chem. 2014;146: 174–180. doi: 10.1016/j.foodchem.2013.09.063 24176329 10.1016/j.foodchem.2013.09.063

[6] Mendonça ICG, Porto ICCM, Nascimento TG, de Souza NS, Oliveira JMS, Arruda RES, et al Brazilian red propolis: phytochemical screening, antioxidant activity and effect against cancer cells. BMC Complement Altern Med. BioMed Central; 2015;15: 357 doi: 10.1186/s12906-015-0888-9 26467757 10.1186/s12906-015-0888-9 PMC4604764

[7] Piccinelli AL, Lotti C, Campone L, Cuesta-Rubio O, Campo Fernandez M, Rastrelli L. Cuban and brazilian red propolis: botanical origin and comparative analysis by high-performance liquid chromatography-photodiode array detection/ electrospray ionization tandem mass spectrometry. J Agric Food Chem. 2011;59: 6484–6491. doi: 10.1021/jf201280z 21598949 10.1021/jf201280z

[8] Frozza COS, Garcia CSC, Gambato G, Souza MDO, Salvador M, Moura S, et al Chemical characterization, antioxidant and cytotoxic activities of Brazilian red propolis. Food Chem Toxicol. Elsevier Ltd; 2013;52: 137–42. doi: 10.1016/j.fct.2012.11.013 23174518 10.1016/j.fct.2012.11.013

[9] Regueira MS, Tintino SR, da Silva ARP, Costa MS, Boligon AA, Matias EFF, et al Seasonal variation of Brazilian red propolis: Antibacterial activity, synergistic effect and phytochemical screening. Food Chem Toxicol. 2017;107: 572–580. doi: 10.1016/j.fct.2017.03.052 28359875 10.1016/j.fct.2017.03.052

[10] Silva RPD, Machado BAS, Barreto GA, Costa SS, Andrade LN, Amaral RG, et al Antioxidant, antimicrobial, antiparasitic, and cytotoxic properties of various Brazilian propolis extracts. PLoS One. 2017;12: e0172585 doi: 10.1371/journal.pone.0172585 28358806 10.1371/journal.pone.0172585 PMC5373518

[11] Neves MVM, Silva TMS, Lima EO, Cunha EVL, Oliveira EJ. Isoflavone formononetin from red propolis acts as a fungicide against *Candida* sp. Brazilian J Microbiol. 2016;47: 159–166. doi: 10.1016/j.bjm.2015.11.009 26887239 10.1016/j.bjm.2015.11.009 PMC4822756

[12] Nascimento TG, Silva PF, Azevedo LF, Rocha LG, Moraes Porto ICC, Moura TFAL, et al Polymeric Nanoparticles of Brazilian red propolis extract: preparation, characterization, antioxidant and leishmanicidal activity. Nanoscale Res Lett. 2016;11: 301 doi: 10.1186/s11671-016-1517-3 27316742 10.1186/s11671-016-1517-3 PMC4912519

[13] Begnini KR, Moura de Leon PM, Thurow H, Schultze E, Campos VF, Martins Rodrigues F, et al Brazilian red propolis induces apoptosis-like cell death and decreases migration potential in bladder cancer cells. Evidence-Based Complement Altern Med. 2014;2014: 1–13. doi: 10.1155/2014/639856 25530785 10.1155/2014/639856 PMC4235187

[14] Frozza COS, Santos DA, Rufatto LC, Minetto L, Scariot FJ, Echeverrigaray S, et al Antitumor activity of Brazilian red propolis fractions against Hep-2 cancer cell line. Biomed Pharmacother. 2017;91: 951–963. doi: 10.1016/j.biopha.2017.05.027 28514834 10.1016/j.biopha.2017.05.027

[15] Bueno-Silva B, Koo H, Falsetta ML, Alencar SM, Ikegaki M, Rosalen PL. Effect of neovestitol—vestitol containing Brazilian red propolis on accumulation of biofilm in vitro and development of dental caries in vivo. Biofouling. 2013;29: 1233–1242. doi: 10.1080/08927014.2013.834050 24099330 10.1080/08927014.2013.834050 PMC3855307

[16] Bueno-Silva B, Kawamoto D, Ando-Suguimoto ES, Alencar SM, Rosalen PL, Mayer MPA. Brazilian red propolis attenuates inflammatory signaling cascade in lps-activated macrophages. PLoS One. 2015;10: e0144954 doi: 10.1371/journal.pone.0144954 26660901 10.1371/journal.pone.0144954 PMC4684384

[17] Bueno-Silva B, Kawamoto D, Ando-Suguimoto ES, Casarin RCV, Alencar SM, Rosalen PL, et al Brazilian red propolis effects on peritoneal macrophage activity: nitric oxide, cell viability, pro-inflammatory cytokines and gene expression. J Ethnopharmacol. 2017;207: 100–107. doi: 10.1016/j.jep.2017.06.015 28624363 10.1016/j.jep.2017.06.015

[18] Cavendish RL, Santos JS, Belo Neto R, Paixão AO, Oliveira JV, Araujo ED, et al Antinociceptive and anti-inflammatory effects of Brazilian red propolis extract and formononetin in rodents. J Ethnopharmacol. 2015;173: 127–133. doi: 10.1016/j.jep.2015.07.022 26192808 10.1016/j.jep.2015.07.022

[19] Corrêa FRS, Schanuel FS, Moura-Nunes N, Monte-Alto-Costa A, Daleprane JB. Brazilian red propolis improves cutaneous wound healing suppressing inflammation-associated transcription factor NFκB. Biomed Pharmacother. 2017;86: 162–171. doi: 10.1016/j.biopha.2016.12.018 27978495 10.1016/j.biopha.2016.12.018

[20] Alday-Provencio S, Diaz G, Rascon L, Quintero J, Alday E, Robles-Zepeda R, et al Sonoran propolis and some of its chemical constituents inhibit in vitro growth of *Giardia lamblia* trophozoites. Planta Med. 2015;81: 742–747. doi: 10.1055/s-0035-1545982 26008200 10.1055/s-0035-1545982

[21] Freitas SF, Shinohara L, Sforcin JM, Guimarães S. In vitro effects of propolis on *Giardia duodenalis* trophozoites. Phytomedicine. 2006;13: 170–175. doi: 10.1016/j.phymed.2004.07.008 16428024 10.1016/j.phymed.2004.07.008

[22] Starzyk J, Scheller S, Szaflarski J, Moskwa M, Stojko A. Biological properties and clinical application of propolis. II. Studies on the antiprotozoan activity of ethanol extract of propolis. Arzneimittelforschung. 1977;27: 1198–9. 302709

[23] Alvarez-Sanchez ME, Villalpando JL, Quintas-Granados LI, Arroyo R. Polyamine transport and synthesis in Trichomonas vaginalis: potential therapeutic targets. Curr Pharm Des. 2017;23 doi: 10.2174/1381612823666170703162754 28671057 10.2174/1381612823666170703162754

[24] Cudmore SL, Delgaty KL, Hayward-McClelland SF, Petrin DP, Garber GE. Treatment of infections caused by metronidazole-resistant *Trichomonas vaginalis*. Clin Microbiol Rev. 2004;17: 783–93, table of contents. doi: 10.1128/CMR.17.4.783-793.2004 15489348 10.1128/CMR.17.4.783-793.2004 PMC523556

[25] Fischer G, Paulino N, Marcucci MC, Siedler BS, Munhoz LS, Finger PF, et al Green propolis phenolic compounds act as vaccine adjuvants, improving humoral and cellular responses in mice inoculated with inactivated vaccines. Mem Inst Oswaldo Cruz. 2010;105: 908–913. doi: 10.1590/S0074-02762010000700012 21120362 10.1590/s0074-02762010000700012

[26] Ashry el SH, Ahmad TA. The use of propolis as vaccine’s adjuvant. Vaccine. 2012;31: 31–9. doi: 10.1016/j.vaccine.2012.10.095 23137844 10.1016/j.vaccine.2012.10.095

[27] Sforcin JM, Bankova V. Propolis: is there a potential for the development of new drugs? J Ethnopharmacol. 2011;133: 253–60. doi: 10.1016/j.jep.2010.10.032 20970490 10.1016/j.jep.2010.10.032

[28] Biscaia D, Ferreira SRS. Propolis extracts obtained by low pressure methods and supercritical fluid extraction. J Supercrit Fluids. 2009;51: 17–23. doi: 10.1016/J.SUPFLU.2009.07.011

[29] Bankova V, Popova M, Trusheva B. Propolis volatile compounds: chemical diversity and biological activity: a review. 2014;8: 1–8. doi: 10.1186/1752-153X-8-28 24812573 10.1186/1752-153X-8-28 PMC4014088

[30] Suzuki S, Kazuhiro Amano, Suzuki K. Effect of propolis volatiles from a stingless honeybee (Apidae: Meliponinae) on the immune system of elderly residents in a nursing home. Int J Indust Entomol. 2009;19: 193–197.

[31] Abikim G, Yimit R, Tursunay A, Aerziguli T, Mutallip A. Effect of essential oils extracted from Xingjiang propolis on cell proliferation, cell cycle progression and apoptosis in human colorectal cancer cell line HTC-116. Shijie Huaren Xiaohua Zazhi. 2011;19: 1469–1475.

[32] Diamond LS. The establishment of various trichomonads of animals and man in axenic cultures. J Parasitol. 1957;43: 488–90. 13463700

[33] Hübner DPG, Vieira PB, Frasson AP, Menezes CB, Senger FR, Silva GNS, et al Anti-*Trichomonas vaginalis* activity of betulinic acid derivatives. Biomed Pharmacother. 2016;84: 476–484. doi: 10.1016/j.biopha.2016.09.064 27685791 10.1016/j.biopha.2016.09.064

[34] Droppa-Almeida D, Vivas WLP, Kelly K, Silva O, Rezende AFS, Simionatto S, et al Recombinant CP40 from *Corynebacterium pseudotuberculosis* confers protection in mice after challenge with a virulent strain. Vaccine. 2016;34: 1091–1096. doi: 10.1016/j.vaccine.2015.12.064 26796140 10.1016/j.vaccine.2015.12.064

[35] Lemberkovics E, Kakasy AZ, Héthelyi BE, Simándi B, Böszörményi A, Balázs A, et al Gas chromatography for analysis of essential oils. Characteristics of essential oil of *Dracocephalum* species and the influence of extraction method on its composition. Acta Pharm Hung. 2007;77: 19–27. 17518109

[36] Oliveira A, França H, Kuster R, Teixeira L, Rocha L. Chemical composition and antibacterial activity of Brazilian propolis essential oil. J Venom Anim Toxins Incl Trop Dis. 2010;16: 121–130. doi: 10.1590/S1678-91992010005000007

[37] Quijano CE, Salamanca G, Pino JA. Aroma volatile constituents of Colombian varieties of mango (*Mangifera indica* L.). Flavour Fragr J. 2007;22: 401–406. doi: 10.1002/ffj.1812

[38] Shellie RA, Marriott PJ. Comprehensive two-dimensional gas chromatography-mass spectrometry analysis of *Pelargonium graveolens* essential oil using rapid scanning quadrupole mass spectrometry. Analyst. 2003;128: 879 doi: 10.1039/b304371a

[39] Yayli N., Yasar A., Gülec C., Usta A., Kolayli S., Coskuncelebi K., et al Composition and antimicrobial activity of essential oils from *Centaurea sessilis* and *Centaurea armena*. Phytochemistry. 2005;66: 1741–1745. doi: 10.1016/j.phytochem.2005.04.006 16050993 10.1016/j.phytochem.2005.04.006

[40] Boussaada O, Ammar S, Saidana D, Chriaa J, Chraif I, Daami M, et al Chemical composition and antimicrobial activity of volatile components from capitula and aerial parts of *Rhaponticum acaule* DC growing wild in Tunisia. Microbiol Res. 2008;163: 87–95. doi: 10.1016/j.micres.2007.02.010 17482441 10.1016/j.micres.2007.02.010

[41] Isidorov VA, Vinogorova VT, Rafalowski K. HS-SPME analysis of volatile organic compounds of coniferous needle litter. Atmos Environ. 2003;37: 4645–4650. doi: 10.1016/J.ATMOSENV.2003.07.005

[42] Yáñez X, Pinzón ML, Solano F, Sánchez LR. Chemical composition of the essential oil of *Psidium caudatum* McVaugh. Molecules. 2002;7: 712–716. doi: 10.3390/70900712

[43] Tzakou O, Couladis M, Slavkovska V, Mimica-Dukic N, Jancic R. The essential oil composition of *Salvia brachyodon* Vandas. Flavour Fragr J. 2003;18: 2–4. doi: 10.1002/ffj.1132

[44] Fokialakis N, Magiatis P, Mitaku S. Essential oil constituents of *Valeriana italica* and *Valeriana tuberosa*. Stereochemical and conformational study of 15-acetoxyvaleranone. Z Naturforsch C. 2002;57: 791–6. 12440713 10.1515/znc-2002-9-1006

[45] Flach A, Dondon RC, Singer RB, Koehler S, Amaral MDCE, Marsaioli AJ. The chemistry of pollination in selected Brazilian Maxillariinae orchids: floral rewards and fragrance. J Chem Ecol. 2004;30: 1045–56. 15274447 10.1023/b:joec.0000028466.50392.ed

[46] Flamini G, Cione PL, Morelli I. Composition of the essential oils and in vivo emission of volatiles of four *Lamium* species from Italy: *L*. *purpureum*, *L*. *hybridum*, *L*. *bifidum* and *L*. *amplexicaule*. Food Chem. 2005;91: 63–68. doi: 10.1016/J.FOODCHEM.2004.05.047

[47] Aligiannis N, Kalpoutzakis E, Chinou IB, Mitakou S, Gikas E, Tsarbopoulos A. Composition and antimicrobial activity of the essential oils of five taxa of *Sideritis* from greece. American Chemical Society; 2001; doi: 10.1021/JF001018W 10.1021/jf001018w 11262034

[48] Hazzit M, Baaliouamer A, Faleiro ML, Miguel MG. Composition of the essential oils of *Thymus* and *Origanum* species from Algeria and their antioxidant and antimicrobial activities. J Agric Food Chem. 2006;54: 6314–6321. doi: 10.1021/jf0606104 16910725 10.1021/jf0606104

[49] Bankova V, Boudourova-Krasteva G, Popov S, Sforcin JM, Funari SRC. Seasonal variations in essential oil from brazilian propolis. J Essent Oil Res. 1998;10: 693–696. doi: 10.1080/10412905.1998.9701012

[50] Ioshida MDM, Young MCM, Lago JHG. Chemical composition and antifungal activity of essential oil from Brazilian propolis. J Essent Oil Bear Plants. 2010;13: 633–637. doi: 10.1080/0972060X.2010.10643873

[51] López BGC, Schmidt EM, Eberlin MN, Sawaya ACHF. Phytochemical markers of different types of red propolis. Food Chem. 2014;146: 174–180. doi: 10.1016/j.foodchem.2013.09.063 24176329 10.1016/j.foodchem.2013.09.063

[52] Freires IA, de Alencar SM, Rosalen PL. A pharmacological perspective on the use of Brazilian red propolis and its isolated compounds against human diseases. Eur J Med Chem. 2016;110: 267–279. doi: 10.1016/j.ejmech.2016.01.033 26840367 10.1016/j.ejmech.2016.01.033

[53] Hyldgaard M, Mygind T, Meyer RL. Essential oils in food preservation: mode of action, synergies, and interactions with food matrix components. Front Microbiol. 2012;3: 12 doi: 10.3389/fmicb.2012.00012 22291693 10.3389/fmicb.2012.00012 PMC3265747

[54] Calleja MA, Vieites JM, Montero-Meterdez T, Torres MI, Faus MJ, Gil A, et al The antioxidant effect of β-caryophyllene protects rat liver from carbon tetrachloride-induced fibrosis by inhibiting hepatic stellate cell activation. Br J Nutr. 2013;109: 394–401. doi: 10.1017/S0007114512001298 22717234 10.1017/S0007114512001298

[55] Palmer-Young EC, Veit D, Gershenzon J, Schuman MC. The Sesquiterpenes(E)-ß-Farnesene and (E)-α-Bergamotene quench ozone but fail to protect the wild tobacco *Nicotiana attenuata* from ozone, UVB, and Drought Stresses. PLoS One. 2015;10: e0127296 doi: 10.1371/journal.pone.0127296 26030663 10.1371/journal.pone.0127296 PMC4452144

[56] Li HQ, Liu QZ, Liu ZL, Du SS, Deng ZW. Chemical composition and nematicidal activity of essential oil of *Agastache rugosa against Meloidogyne incognita*. Molecules. 2013;18: 4170–4180. doi: 10.3390/molecules18044170 23571530 10.3390/molecules18044170 PMC6270543

[57] Trusheva B, Popova M, Bankova V, Simova S, Marcucci MC, Miorin PL, et al Bioactive constituents of Brazilian red propolis. Evid Based Complement Alternat Med. 2006;3: 249–54. doi: 10.1093/ecam/nel006 16786055 10.1093/ecam/nel006 PMC1475931

[58] Wachter B, Syrowatka M, Obwaller A, Walochnik J. In vitro efficacy of curcumin on *Trichomonas vaginalis*. Wien Klin Wochenschr. 2014; S32–6. doi: 10.1007/s00508-014-0522-8 24619489 10.1007/s00508-014-0522-8

[59] Zemek J, Valent M, Pódová M, Kosíková B, Joniak D. Antimicrobial properties of aromatic compounds of plant origin. Folia Microbiol (Praha). 1987;32: 421–5. 3121479 10.1007/BF02887573

[60] Omar RMK, Igoli J, Gray AI, Ebiloma GU, Clements C, Fearnley J, et al Chemical characterisation of Nigerian red propolis and its biological activity against *Trypanosoma brucei*. Phytochem Anal. 2016;27: 107–115. doi: 10.1002/pca.2605 26662866 10.1002/pca.2605

[61] Ayres DC, Marcucci MC, Giorgio S. Effects of Brazilian propolis on *Leishmania amazonensis*. Mem Inst Oswaldo Cruz. 2007;102: 215–220. doi: 10.1590/S0074-02762007005000020 17426888 10.1590/s0074-02762007005000020

[62] Silva JW, Droppa-Almeida D, Borsuk S, Azevedo V, Portela RW, Miyoshi A, et al *Corynebacterium pseudotuberculosis* cp09 mutant and cp40 recombinant protein partially protect mice against caseous lymphadenitis. BMC Vet Res. 2014;10: 965 doi: 10.1186/s12917-014-0304-6 25527190 10.1186/s12917-014-0304-6 PMC4297461

[63] Guy B. The perfect mix: recent progress in adjuvant research. Nat Rev Microbiol. 2007;5: 505–17. 17558426 10.1038/nrmicro1681

[64] Roslindawani MN, Syafiqah AS, Jesse FFA, Effendy AW, Zamri-Saad M. Recombinant caseous lymphadenitis vaccine with palm oil as adjuvant enhances the humoral and cell-mediated immune responses in rat model. J Anim Heal Prod. 2016;4: 23.

[65] Dong C. Diversification of T-helper-cell lineages: finding the family root of IL-17-producing cells. Nat Rev Immunol. 2006;6: 329–33. doi: 10.1038/nri1807 16557264 10.1038/nri1807

[66] Gause KT, Wheatley AK, Cui J, Yan Y, Kent SJ, Caruso F. Immunological principles guiding the rational design of particles for vaccine delivery. ACS Nano. 2017;11: 54–68. doi: 10.1021/acsnano.6b07343 28075558 10.1021/acsnano.6b07343

[67] Vishteh A, Thomas I, Imamura T. Eugenol modulation of the immune response in mice. Immunopharmacology. 1986;12: 187–92. Available: http://www.ncbi.nlm.nih.gov/pubmed/3546191 3546191 10.1016/0162-3109(86)90002-0

[68] López-Antón N, Hermann C, Murillo R, Merfort I, Wanner G, Vollmar AM, et al Sesquiterpene lactones induce distinct forms of cell death that modulate human monocyte-derived macrophage responses. Apoptosis. 2007;12: 141–153. doi: 10.1007/s10495-006-0331-2 17080325 10.1007/s10495-006-0331-2

[69] Bezerra GB, Souza LM, Santos AS, Almeida GKM, Souza MTS, Santos SL, et al Hydroalcoholic extract of Brazilian red propolis exerts protective effects on acetic acid-induced ulcerative colitis in a rodent model. Biomed Pharmacother. 2017;85: 687–696. doi: 10.1016/j.biopha.2016.11.080 27955827 10.1016/j.biopha.2016.11.080

[70] Bueno-Silva B, Franchin M, Alves de C F, Denny C, Colón DF, Cunha TM, et al Main pathways of action of Brazilian red propolis on the modulation of neutrophils migration in the inflammatory process. Phytomedicine. 2016;23: 1583–1590. doi: 10.1016/j.phymed.2016.09.009 27823622 10.1016/j.phymed.2016.09.009

[71] Fischer G, Hübner SO, Vargas GD, Vidor T. Imunomodulação pela própolis. Arq Inst Biol. 2008;75: 247–253.

